# Supplementary material for: Two-part predictive modeling for COVID-19 cases and deaths in the U.S
Source: PLoS One. 2024 Jun 6;19(6):e0302324. doi: 10.1371/journal.pone.0302324 (PMC11156282; doi:10.1371/journal.pone.0302324)
Supplement: S1 Table — (DOCX) [file pone.0302324.s001.docx]

**S1 Table. Summary of continuous variables.**

| Name | Data Type | Description (Per County) | Mean ± SD |
| --- | --- | --- | --- |
| Population ($x_{1}$) | Continuous | The county's total population | 119,645 ± 394,155 |
| Primary Care Physicians ($x_{2}$) | Continuous | Proportion of primary care physicians | 0.55 ± 0.35 |
| 65 and Older ($x_{3}$) | Continuous | Proportion of people ages 65 and older | 0.17 ± 0.04 |
| 17 and Younger ($x_{4}$) | Continuous | Proportion of people ages 17 and younger | 0.23 ± 0.03 |
| African-Americans ($x_{5}$) | Continuous | Proportion of African-Americans | 0.10 ± 0.15 |
| Indian-Americans and Native-Alaskans ($x_{6}$) | Continuous | Proportion of Indian-Americans and Native Alaskans | 0.02 ± 0.07 |
| Asians and Asian-Americans ($x_{7}$) | Continuous | Proportion of Asians and Asian-Americans | 0.02 ± 0.03 |
| Hawaiians and Pacific-Islanders ($x_{8}$) | Continuous | Proportion of Hawaiians and Pacific-Islanders | 0.001 ± 0.004 |
| Hispanic ($x_{9}$) | Continuous | Proportion of Hispanics | 0.10 ± 0.14 |
| Non-Hispanic White ($x_{10}$) | Continuous | Proportion of Non-Hispanic White | 0.76 ± 0.20 |
| Rural ($x_{11}$) | Continuous | Proportion of people who live in rural communities | 0.55 ± 0.31 |
| Below Poverty ($x_{12}$) | Continuous | Proportion of people who live below poverty | 0.16 ± 0.06 |
| Overcrowding ($x_{13}$) | Continuous | Proportion of people living in housing where the number of occupants exceeds the unit’s capacity | 0.02 ± 0.02 |
| Mean Temperature ($x_{14}$) | Continuous | Average temperature recorded | 60.8 ± 14.5 |
| Cases ($x_{15}$) | Continuous | Cumulative COVID-19 cases | 1890 ± 9219 |
| Deaths ($x_{16}$) | Continuous | Cumulative deaths due to COVID-19 | 52.4 ± 454 |
