# Supplementary material for: Two-part predictive modeling for COVID-19 cases and deaths in the U.S
Source: PLoS One. 2024 Jun 6;19(6):e0302324. doi: 10.1371/journal.pone.0302324 (PMC11156282; doi:10.1371/journal.pone.0302324)
Supplement: S2 Table — (DOCX) [file pone.0302324.s002.docx]

**S2 Table. Summary of categorical variables.**

| Name | Data Type | Description | Count |
| --- | --- | --- | --- |
| State | Categorical | U.S. States and District of Columbia | Texas = 2,268  Georgia = 1,517  Virginia = 1,301  Kentucky = 1,120  Missouri = 1,087 |
| Stay-at-Home Effective | Categorical | A binary variable indicating whether a county and/or state has implemented a stay-at-home order | Yes = 23,252  No = 7,008 |
