# Supplementary figures and images for: Two-part predictive modeling for COVID-19 cases and deaths in the U.S
Source: PLoS One. 2024 Jun 6;19(6):e0302324. doi: 10.1371/journal.pone.0302324 (PMC11156282; doi:10.1371/journal.pone.0302324)

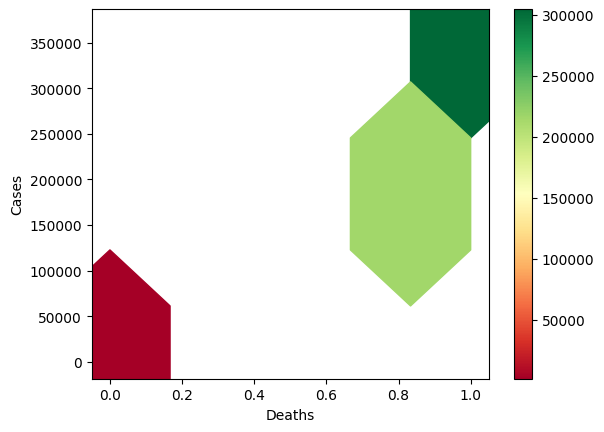

Supplement: S1 Fig — (TIFF) [file pone.0302324.s003.tiff]

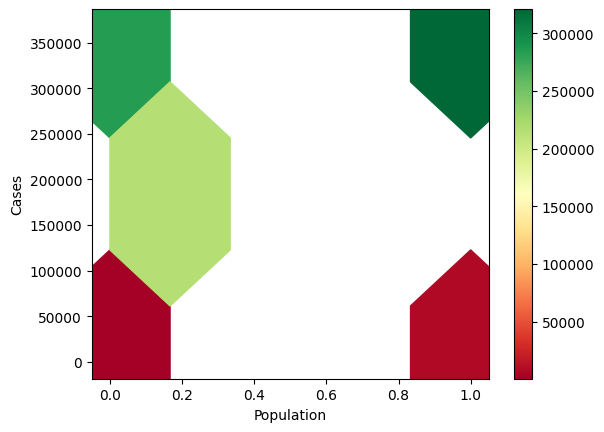

Supplement: S2 Fig — (TIFF) [file pone.0302324.s004.tiff]

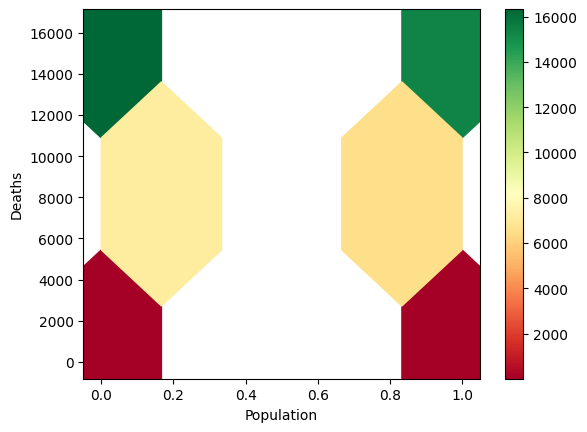

Supplement: S3 Fig — (TIFF) [file pone.0302324.s005.tiff]

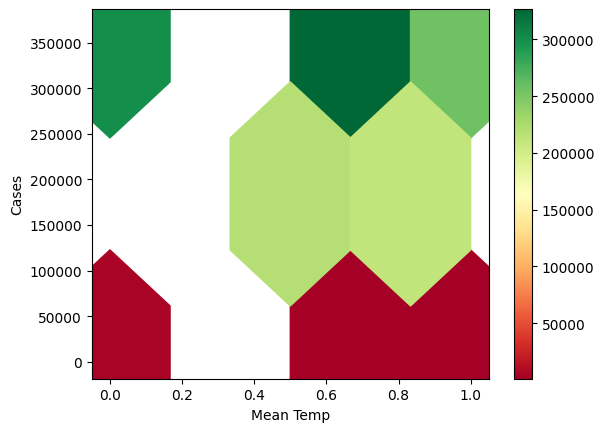

Supplement: S4 Fig — (TIFF) [file pone.0302324.s006.tiff]

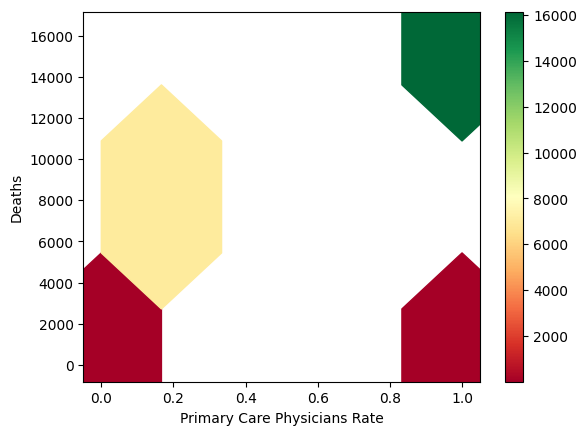

Supplement: S5 Fig — (TIFF) [file pone.0302324.s007.tiff]
