# Supplementary material for: Two-part predictive modeling for COVID-19 cases and deaths in the U.S
Source: PLoS One. 2024 Jun 6;19(6):e0302324. doi: 10.1371/journal.pone.0302324 (PMC11156282; doi:10.1371/journal.pone.0302324)
Supplement: Attachment — Submitted filename: REV_PONE-D-23-42948.pdf [file pone.0302324.s008.pdf]

REVIEW REPORT OF THE PAPER TITLED: "TWO-PART PREDICTIVE MODELING FOR COVID-19 CASES AND DEATHS IN THE U.S"

The paper illustrates a two-step approach to provide predictions of cases and deaths concerning COVID-19 data referred to US. The authors propose using logistic regression and standard machine learning models. Comparisons among predictive models are based on standard metrics.

The authors have to account for the following major concerns:

- The proposed model is generally named the multiple linear logistic regression model (so the word binary is generally avoided).
- Avoid writing "etc..." in the abstract.
- Introduction: also specify that generally, a zero-inflated model can be considered instead of using a two-step approach. Add also benefits and drawbacks of the two approaches.

- Line 24 (and line 310): replace the generic URL of Kaggle with the link relative to the data used in the paper (the data file). Maybe it could be placed in a note. The link in the concluding remarks is not needed.
- On page 3, check the text in line 93.
- It is also convenient to report and comment on the partial correlations (see the R package ggm for a related function).
- Please justify the choice of the min-max scaling. Evaluate if a better option is provided by using a Box-Cox or a logistic transformation.
- Line 135: How did you split the data? Did you check the sensitivity of your choice? How?
- Lines 138-139: add proper references for the reported procedures.
- Line 158: add a proper citation for the ROC curve.
- Line 186: a citation for the GAM.
- Line 196: add a comma after x_2i.
- Line 201: add a citation for the R package mgcv.
- Line 214: uniform the text for randomForest since the names of the other packages are written in bold.

- Line 244: replace a semicolon with a dot.
- Caption of Table 4: specify the measure of the time.
- Line 301: delete s in show.
- Maximum likelihood inference for hidden Markov models with parsimonious parametrizations of transition matrices.
