# Supplementary material for: Two-part predictive modeling for COVID-19 cases and deaths in the U.S
Source: PLoS One. 2024 Jun 6;19(6):e0302324. doi: 10.1371/journal.pone.0302324 (PMC11156282; doi:10.1371/journal.pone.0302324)
Supplement: Attachment — Submitted filename: responses_to_reviewers.pdf [file pone.0302324.s009.pdf]

Responses to Referee Comments

We the authors are very thankful to the two reviewers and the Editor for their suggestions and comments, which we believe greatly help improve the presentation of this paper.

Referee 1's Comments

- *The proposed model is generally named the multiple linear logistic regression model (so the word binary is generally avoided).*

  Response: We removed the word binary.

- *Avoid writing "etc..." in the abstract.*

  Response: We rewrote the sentence in the abstract to remove "etc".

- *Introduction: also specify that generally, a zero-inflated model can be considered instead of using a two-step approach. Add also benefits and drawbacks of the two approaches.*

  Response: Different models are best for different research goals. The primary research goal in this study is to analyze the non-zero component of COVID-cases or deaths in the data set. The two-part modeling is easy to implement, and focuses on the non-zero outcomes. This framework separated zero component from non-zero component very well in the first step, according to its high AUC values. In the second step, machine learning methods and smoothing methods had good evaluation metric values. On the other hand, a zero-inflated distribution can be considered as a mixture of two count distributions, and a zero-inflated model treats zero and non-zero outcomes equally, which doesn't agree with the research goal in this study. We added the following "In general, a zero-inflated model can be used when the outcome variable has a large mass at zero. However, different models are best for different research goals. The primary research goal in this study is to analyze the non-zero component of COVID-cases or deaths in the data set. The two-part modeling is easy to implement, can separate the zero outcomes and the non-zero outcomes well, and focuses on the non-zero outcomes. On the other hand, a zero-inflated distribution can be considered as a mixture of two count distributions, and a zero-inflated model treats zero and non-zero outcomes equally." in lines 36 to 43.

- *Line 24 (and line 310): replace the generic URL of Kaggle with the link relative to the data used in the paper (the data file). Maybe it could be placed in a note. The link in the concluding remarks is not needed.*

  Response: The generic URL in line 24 was removed since we already stated the data set was downloaded from Kaggle, it is unnecessary to link the Kaggle website. The generic links for each database were also replaced with relative links that give direct access in Section 3.1.

- *It is also convenient to report and comment on the partial correlations (see the R package ggm for a related function).*

  Response: We checked the ggm package, used the parcor function to generate partial correlations, and visualized it. In the paper, we replaced the original Figure 2 with a new plot in which the left panel is the correlation matrix generated by the ggcorrplot package, and the right panel is the partial correlation matrix generated by the ggm package and the ggcorrplot package. A citation for the ggm package was added as [9]. Brief explanation about partial correlations was added in the paper as following: "The right panel is the partial correlation matrix generated by the **ggm** [9] package and the ggcorrplot package. Unlike Pearson correlation, partial correlation measures pairwise linear

relationship after controlling for other variables. We observed several very strong partial correlations, which especially appeared among racial percentages. For example, the partial correlations between percentages of African Americans (x_5) and percentages of Hispanic (x_9) residents, percentage of Indian-Americans and Native Alaskans residents, and percentage of non-Hispanic White residents (x_{10})."

- *Please justify the choice of the min-max scaling. Evaluate if a better option is provided by using a Box-Cox or a logistic transformation.*

  Response: The min-max scaling is a commonly used scaling method. It will dampen the influence of outliers and bring the range of each predictor to be within 0 and 1. Meanwhile, it won't change the shape of the distribution of each predictor. Box-Cox transformation is used to attain an approximately normally distributed predictor. In this study, we don't need to have features to follow a normal distribution. Logistic transformation will scale a variable to be within 0 and 1, but it changes the shape of the original distribution. Due to the extreme skew pattern of COVID-19 cases and deaths, what we care about a lot is to reduce the influence of outliers in model fitting. Besides, in this study, we proposed a two-part GAM model for its good interpretability of the relationship between features and the outcome variable. If we apply Box-Cox transformation or logistic transformation to a predictor, the interpretation will be hard or at least less straightforward. We added the justification for min-max scaling in the paper as "The min-max scaling is a commonly used scaling method. It will dampen the influence of outliers and bring the range of each predictor to be within 0 and 1. Meanwhile, it won't change the shape of the distribution of each predictor, which makes it easier to interpret the relationship between the x's and the outcome of interest than some method like Box-Cox transformation." in lines 161 to 165.

- *Line 135: How did you split the data? Did you check the sensitivity of your choice? How?*

  Response: We shuffled the data set and randomly select 80% data points to form a training set and use the remaining 20% to form a test set. We are in a data-rich situation: the total number of observations is about 30,000. Both training set and test set have a large sample size. The 80% vs 20% is a typical rule used in machine learning workflow. We prefer using this rule instead of something like 50% vs 50% splitting because we tried models like ANN with two layers, which was complex and required a lot of parameter tuning, and it is better to use more data points to train complex models in order to reduce the variance of model fitting. Moreover, we used 5-fold cross validation with the training set, which helps with the accuracy of prediction error estimation. We believe that the splitting is valid and we didn't check the sensitivity of this splitting method.

  We re-wrote the sentence in line 173: "Before modeling, we split the data into training set (80%) and test set (20%).", which was included in the previously submitted version of this paper as: "Before modeling, the data set was randomly shuffled first and then split into a training set 80% vs a test set 20%."

- *Lines 138-139: add proper references for the reported procedures.*

  Response: We added a citation for the book "Pattern Recognition and Neural Networks" as [12] and a citation for the paper "Fast stable restricted maximum likelihood and marginal likelihood estimation of semiparametric generalized linear models" as [13], which contain the background information about implementing the procedure described in Lines 178 - 179 to regression tasks.

- *Line 158: add a proper citation for the ROC curve.*

  Response: We added a citation for the paper "An Introduction to ROC Analysis" as [14] in Line 200.

- *Line 186: a citation for the GAM.*

  Response: We added a citation for the book "Generalized Additive Models" as [20] in Line 228.

- *Line 196: add a comma after x_{2i}.*

  Response: We added the comma after x_{2i} in line 237.

- *Line 201: add a citation for the R package mgcv.*

  Response: We added a citation for the book "Generalized Additive Models: An Introduction with R" as [21] in Line 243.

- *Line 214: uniform the text for randomForest since the names of the other packages are written in bold.*

  Response: We bolded the text for randomForest.

- *Line 244: replace a semicolon with a dot.*

  Response: We replaced the semicolon with a dot.

- *Caption of Table 4: specify the measure of the time.*

  Response: We specified the measure of time (in minutes).

- *Line 301: delete s in show.*

  Response: We deleted the s in show.

Referee 2's Comments

- *It appears that there might not be explicit mention of checking for collinearity, for example, through methods like the Variance Inflation Factor (VIF). Assessing collinearity can be crucial in ensuring the robustness of regression models.*

  Response: We used the vif function in the **car** package to compute the generalized variance-inflation factor (GVIF). In the car package, generalized variance-inflation factors are computed when some term has more than 1 degree of freedom, which is the case of this study because the predictor "state" has 50 degree of freedoms. After adjusting for the degree of freedom, we found that percentage of black residents, percentage of Hispanic residents, and percentage of non Hispanic white residents have GVIF values larger than 10. PCA of these racial percentage variables could be done in order to reduce GVIF values, but this may make it more difficult to interpret the relationship between the outcome and each racial percentage variable.

  We added the following: "We used the vif function in the **car** package [10] to compute the generalized variance-inflation factor (GVIF) [11]. In the car package, generalized variance-inflation factors are computed when some term has more than 1 degree of freedom, which is the case of this study because the predictor "state" has 50 degree of freedoms. After adjusting for the degree of freedom, we found that percentage of black residents, percentage of Hispanic residents, and percentage of non Hispanic white residents have GVIF values larger than 10." in lines 135 to 141.

- *Given that your model incorporates population, total infected cases, and total deaths, it appears these variables may have interdependencies. To enhance the robustness of your findings, it is valuable to conduct a sensitivity analysis. This would allow you to explore how changes in variables may impact the model results and provide insights into the stability of your conclusions.*

  Response: We did 2 sets of sensitivity analysis using the sensitivity package in Python. The model we evaluated is the optimal model: XGBoost. The predictors we chose to vary were the top 3 predictors in the variable importance ranking plots shown in Figure 5. First, we considered cases as the response variable and chose deaths, population, and mean temp as the 3 predictors to vary. Specifically, we chose 0 and another 4 quantiles in the non-zero component in deaths, chose 5 quantiles in population, and 5 quantiles in mean temp. For other predictors, we fixed it at its mean value if the predictor is continuous; if the predictor is categorical, then we fixed the predictor at the category with the most observations. This gave us 125 combinations and we evaluated the XGBoost model with each of the 125 combinations. Heatmaps were created for cases vs deaths, cases vs population, and cases vs mean temp. In the second set of sensitivity analysis, we considered deaths as the response variable, and chose 0 and another 4 quantiles in the non-zero component in cases, chose 5 quantiles in primary care physicians rate, 5 quantiles in population. Other parts were the same as the first set of sensitivity analysis.

  A new paragraph was added in Section 4.6 as following:

  To enhance the findings of the XGBoost model, we did 2 sets of sensitivity analysis using the **sensitivity** package in Python [27]. The predictors we chose to vary were the top 3 predictors in the variable importance ranking plots shown in Fig 5. First, we considered cases as the response variable and chose deaths, population, and mean temp as the 3 predictors to vary. Specifically, we chose 0 and another 4 quantiles in the non-zero component in deaths, chose 5 quantiles in population, and 5 quantiles in mean temp. For other predictors, we fixed it at its mean value if the predictor is continuous; if the predictor is categorical, then we fixed the predictor at the category with the most observations. This gave us 125 combinations and we evaluated the XGBoost model with each of the 125 combinations. Heatmaps were created for cases vs deaths, cases vs population, and cases vs mean temp. In the second set of sensitivity analysis, we considered deaths as the response variable, and chose 0 and another 4 quantiles in the non-zero component in cases, chose 5 quantiles in primary care physicians rate, 5 quantiles in population. Other parts were the same as the first set of sensitivity analysis.

  The information in the following heatmap is similar to that shown in Fig 2. However, in this heatmap, we saw that as the number of COVID-19 cases was large, most numbers of deaths were predicted to be around the median value of the distribution of deaths. Another five heatmaps were included in the Appendix.

- *The manuscript must be checked for punctuation, and errors in grammar.*

  Response: We fixed all grammar and punctuation errors that we could find.

- *There should be no citation in the abstract section according to journal's guidelines. It seems you cited your data source in the abstract.*

  Response: We removed the citation of Kaggle in the abstract section.

- *The authors have used the words "COVID-19" and "COVID" inconsistently. I think it is better to use "COVID-19" throughout the paper.*

  Response: We replaced all instances of "COVID" with "COVID-19".

- *Line 142: there is a small typo. "as least" should be corrected to "at least".*

  Response: We corrected the typo "as" with "at".

- *Line 192: there is a small punctuation error. The punctuation after "function" should be corrected to ",".*

  Response: We resolved the punctuation error by replacing the period with a comma.

- *Line 267: there should be "." after "Fig 4".*

  Response: The "." was added after "Fig 4".

- *I noticed that present tense was consistently used in the Results and Materials and Methods sections. For example in line 231: "We find that the optimal mtry to be 9 predictors for both of our responses" or in line 271: "We find that the area under the curve (AUC) to be 0.93 and 0.95 for cases and deaths, respectively.". It's generally recommended to use the past tense when describing findings.*

  Response: We changed the Introduction, Materials and Methods, and Results sections using past-tense wording whenever we were talking about findings.

- *I noticed that while some equations in the manuscript are numbered, there are instances where equations are not assigned numbers. I recommend ensuring consistent equation numbering throughout the article.*

  Response: We added equation labels to Section 3.4 line 159, Section 3.5.1 line 193, Section 4.1 line 316 and line 318.

- *Line 142: there is a small typo in "Fig 5 rank features in the two-part XGBoost model according to their feature importance value". "rank" should be corrected to "ranks".*

  Response: We think the referee meant Line 287 for this error. We corrected "rank" to "ranks".

- *line 296: "One advantage of a GAM model when compared with machine learning methods is its statistical inference and interpretation. For example, the R package mgcv will generate summary statistics such as p-value for each predictor." should be moved to Materials and Methods Section.*

  Response: We deleted the two sentences starting from line 296, and added them as line 243 to line 246 in the Materials and Methods Section. Besides, we removed the sentence starting in line 196: "When compared with machine learning models such as ANN and XGboost, a GAM model has better interpretability because of its additive nature." because of it basically means the same thing and it is redundant.

References

[1] McNeil DG. The U.S. now leads the world in confirmed coronavirus cases. The New York Times. 2020.

[2] Fox GC, von Laszewski, G F Wang, Pyne S. AICov: an integrative deep learning framework for COVID-19 forecasting with population covariates. Journal of Data Science. 2021;19(2):293–313. doi:10.6339/21-JDS1007.

[3] Cai K, He W, Yi GY. COVID-19 fatality: a cross-sectional study using adaptive lasso penalized sliced inverse regression. Journal of Data Science. 2022;18(3):483–495. doi:10.6339/JDS.202007_18(3).0015.

[4] Gopal R, Han X, Yaraghi N. Compress the curve: a cross-sectional study of variations in COVID-19 infections across California nursing homes. BMJ Open. 2021;11(1).

[5] Ojinnaka CO, Adepoju OE, Burgess AV, Woodard L. Factors associated with COVID-Related mortality: the case of Texas. Journal of Racial and Ethnic Health Disparities. 2021;8:1505–1510.

[6] Li Y, Temkin-Greener H, Gao S, Cai X. COVID-19 infections and deaths among Connecticut nursing home residents: facility correlates. Journal of the American Geriatrics Society. 2020;68(9):1899–1906.

[7] Duncan I, Loginov M, Ludkovski M. Testing alternative regression frameworks for predictive modeling of health care costs. North American Actuarial Journal. 2015;20:1–23. doi:10.1080/10920277.2015.1110491.

[8] Kassambara A, Patil I. ggcorrplot: Visualization of a Correlation Matrix using 'ggplot2'; 2023. Available from: https://CRAN.R-project.org/package=ggcorrplot.

[9] Marchetti MG, Drton M, Sadeghi K. ggm: Graphical Markov Models with Mixed Graphs; 2024. Available from: https://CRAN.R-project.org/package=ggm

[10] Fox J, Weisberg S. An R Companion to Applied Regression. 3rd ed. Thousand Oaks, CA: Sage; 2019. Available from: https://socialsciences.mcmaster.ca/jfox/Books/Companion/.

[11] Fox J, Monette G. Generalized collinearity diagnostics. Journal of the American Statistical Association. 1992; 87: 178–183.

[12] Ripley BD. Pattern Recognition and Neural Networks. Cambridge University Press; 1996 doi:10.1017/CBO9780511812651.

[13] Wood SN Fast stable restricted maximum likelihood and marginal likelihood estimation of semiparametric generalized linear models. Journal of the Royal Statistical Society (B). 2011; 73(1): 3–36.

[14] Fawcett T. An introduction to ROC analysis. Pattern Recognition Letters. 2006; 27(8):861–874. doi:10.1016/j.patrec.2005.10.010.

[15] Aggarwal CC, editor. Data Classification: Algorithms and Applications. Yorktown Heights, NY, USA: CRC Press; 2014.

[16] Venables WN, Ripley BD. Modern Applied Statistics with S. 4th ed. New York: Springer; 2002. Available from: https://www.stats.ox.ac.uk/pub/MASS4/.

[17] Kuhn M. Building predictive models in R using the caret package. Journal of Statistical Software. 2008;28(5):1–26. doi:10.18637/jss.v028.i05.

[18] Paszke A, Gross S, Massa F, Lerer A, Bradbury J, Chanan G, et al. PyTorch: An Imperative Style, High-Performance Deep Learning Library. In: Advances in Neural Information Processing Systems 32; 2019. p. 8024–8035.

[19] Buitinck L, Louppe G, Blondel M, Pedregosa F, Mueller A, Grisel O, et al. API design for machine learning software: experiences from the scikit-learn project. In: ECML PKDD Workshop: Languages for Data Mining and Machine Learning; 2013. p. 108–122.

[20] Hastie T, Tibshirani R. Generalized Additive Models. 1st ed. Chapman and Hall/CRC; 1990.

[21] Wood SN. Generalized Additive Models: An Introduction with R. 2nd ed. Chapman and Hall/CRC; 2017.

[22] Breiman L. Random forests. Machine Learning. 2001;45:5–32.

[23] Liaw A, Wiener M. Classification and regression by randomForest. R News. 2002;2(3):18–22.

[24] Hastie T, Tibshirani R, Friedman J. The Elements of Statistical Learning: Data mining, Inference and Prediction. 2nd ed. New York, NY, USA: Springer; 2009.

[25] Chen T, Guestrin C. XGboost: a scalable tree boosting system. In: Proceedings of the 22nd ACM SIGKDD International Conference on Knowledge Discovery and Data Mining; 2016. p. 785–794.

[26] Ruder S. An overview of gradient descent optimization algorithms. CoRR. 2016;abs/1609.04747.

[27] DeRobertis N. Sensitivity Analysis in Python. Available from: `https://pypi.org/project/sensitivity/`.

[28] Levy PSL, Lemeshow S. Sampling of Populations: Methods and Applications. 4th ed. Hoboken, NJ, USA: John Wiley & Sons; 2008.
